# CKS1B as Drug Resistance-Inducing Gene—A Potential Target to Improve Cancer Therapy

**DOI:** 10.3389/fonc.2020.582451

**Published:** 2020-09-25

**Authors:** Wenwen Shi, Qiudi Huang, Jiacui Xie, He Wang, Xiyong Yu, Yi Zhou

**Affiliations:** ^1^Key Laboratory of Molecular Target and Clinical Pharmacology, The State Key Laboratory of Respiratory Disease, School of Pharmaceutical Sciences and The Fifth Affiliated Hospital, Guangzhou Medical University, Guangzhou, China; ^2^Center of Cancer Research, The Second Affiliated Hospital, Guangzhou Medical University, Guangzhou, China

**Keywords:** drug resistance, therapeutic target, cell cycling, human cancers, CKS1B

## Abstract

Cancer is a threat to human health and life. Although previously centered on chemical drug treatments, cancer treatment has entered an era of precision targeted therapy. Targeted therapy entails precise guidance, allowing the selective killing of cancer cells and thereby reducing damage to healthy tissues. Therefore, the need to explore potential targets for tumor treatment is vital. Cyclin-dependent kinase regulatory subunit 1B (CKS1B), a member of the conserved cyclin kinase subunit 1 (CKS1) protein family, plays an essential role in cell cycling. A large number of studies have shown that CKS1B is associated with the pathogenesis of many human cancers and closely related to drug resistance. Here, we describe the current understanding of the cellular functions of CKS1B and its underlying mechanisms, summarize a recent study of CKS1B as a target for cancer treatment and discuss the potential of CKS1B as a therapeutic target.

## Introduction

In recent years, the incidence of cancer has increased annually. According to national cancer statistics released by the National Cancer Center in 2019, in 2015, 3.929 million new malignant tumors were diagnosed in individuals 0−47 years of age in China, corresponding to an incidence of 285.83/100,000. The cumulative incidence of cancer is 21.44%, with lung cancer ranking first in incidence and mortality. Lung cancer is the most common malignant tumor in the world (11.6%) and the leading cause of cancer-related death (18.4%) (1). According to the latest report from the International Agency for Research on Cancer (IARC), there are ~9.63 million lung cancer patients worldwide. In 2018, there were an estimated 2,093,876 new cases of lung cancer and ~1,761,007 deaths due to lung cancer worldwide. Eighty percent of new lung cancer patients have been diagnosed with non–small-cell lung cancer (NSCLC) (2), which exhibits no clinically significant outcomes or symptoms at an early stage. By the time a patient is diagnosed with NSCLC, the optimal treatment period has typically already passed. Seventy-five percent of NSCLC cases are diagnosed as advanced NSCLC, which is associated with a 5-years survival rate of <15% (3, 4). During tumor development, tumor cell growth is uncontrolled, changing the genome, damaging healthy cells due to invasion of the tumor into nearby tissues and transferring tumor cells to distant tissues. In such cases, only non-surgical treatment options are available to patients. Although targeted drug therapy and immunotherapy have good curative effects, chemotherapy remains the preferred treatment for cancer patients in the clinical. However, due to the development of chemotherapy resistance in cancers, the vast majority of patients have a poor prognosis.

## Cyclin-Dependent Kinase Regulatory Subunit 1 (CKS1B)

Malignant tumors are caused by dysregulation of the cell cycle and impaired cell differentiation. Regulation of the cell cycle depends on interactions among cyclins, cyclin-dependent kinase (CDK) and its inhibitors. CKS1B, the protein encoded by the CKS1 gene on human chromosome Iq21, has a molecular weight of 9 kDa and is highly functionally conserved (5). In early coimmunoprecipitation studies, scholars found that CKS1 tightly bound CDK from yeast, human cells, and frog eggs. Therefore, this protein was named cell cycle-dependent protease regulatory subunit (6). CDKs are a family of proteases related to the cell cycle that can degrade CDK substrates and proteins regulated by upstream CDKs. The Cdc kinase subunit (CKS) protein has been isolated and shown to inhibit fission and germination yeast cyclin-dependent kinase 1 (CDK1) gene mutations (7, 8). Two homologs of the yeast CKS gene, CKS1 and CKS2 (9), have been found in mammalian cells. Biochemical and genetic analyses suggest that CKS may play a key role in cell cycle regulation (10).

## Upregulation of Cks1B in Human Cancers

**Figure 1:**
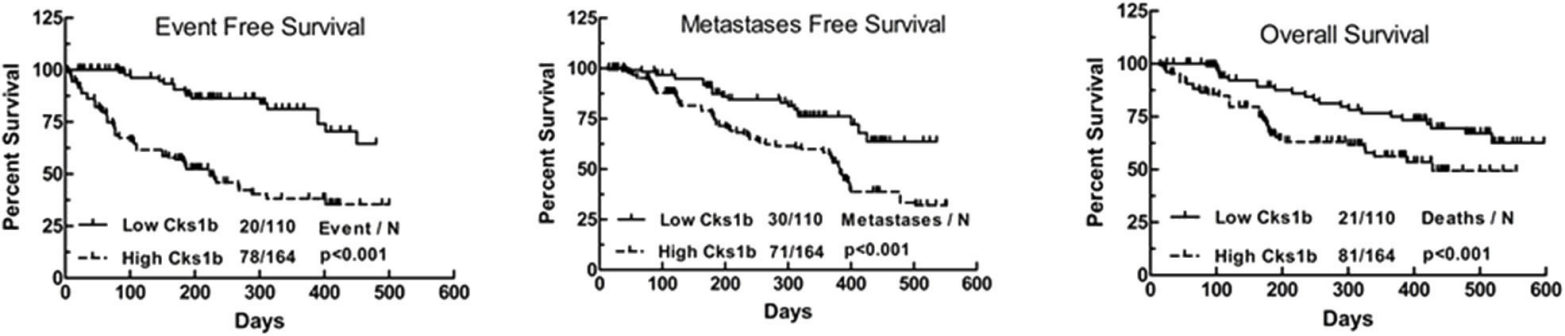
Kaplan–Meier analyses of event-free survival (Left), metastasis-free survival (Medium), and overall survival (Right) revealed inferior outcomes among the 110 lung cancer patients with high Cks1b expression (RPH) relative to the 164 lung cancer patients with low Cks1b expression (IPH).

Various genetic and biochemical experiments in different species have demonstrated the primary functions of CKS1 in normal cell division and growth. The deletion of CKS1 led to mitotic arrest, which eventually decreased cell viability. Due to mitotic retardation, the overexpression of CKS1 produced another abnormal phenotype (11, 12). Functional analysis of CKS1 has shown that (i) CKS1 is essential for maintaining cell viability and that (ii) significant changes in its expression significantly affect the cell division cycle (5). CKS1B, a member of the CKS/Suc1 small protein family, can bind and regulate the function of cyclin-dependent protein kinase catalytic subunits (13, 14). Studies have shown that CKS1B promotes cell growth, invasion, metastasis, and chemical resistance (15, 16). In addition, CKS1B is necessary for normal cell division and growth (5). High CKS1B expression has been shown in many cancers, such as hepatocellular carcinoma (15), colon cancer (17), lung cancer (18), oral squamous cell carcinoma (19), breast cancer (20), and retinoblastoma (RB) (14), among others. Studies have also identified CKS1B as one of 70 high-risk genes whose expression is inversely proportional to the survival of patients newly diagnosed with multiple myeloma (MM) (21). High nuclear expression of CKS1B is also a poor prognostic factor in patients with relapsed/refractory MM (22). These findings provide compelling evidence that CKS1B represents a powerful candidate therapeutic gene (23). Analyses using data from public databases have led to the same conclusion. A study using the public database GENT2 (http://gent2.appex.kr/gent2/) to analyze the expression levels of CKS1B in various cancer types revealed that CKS1B expression levels in brain, colon, bone, ovarian, pancreatic, liver, and lung cancer samples were significantly increased compared with those in healthy tissue samples (24). Kaplan-Meier estimates of event-free survival (left panel), metastasis-free survival (middle panel), and overall survival (right panel) found that recurrent primary human (RPH) lung cancer patients with CKS1B overexpression showed decreased survival compared to that of incipient primary human (IPH) lung cancer patients (Figure 1) (25).

## Cks1B Participates in Regulation of the Cell Cycle

**Figure 2:**
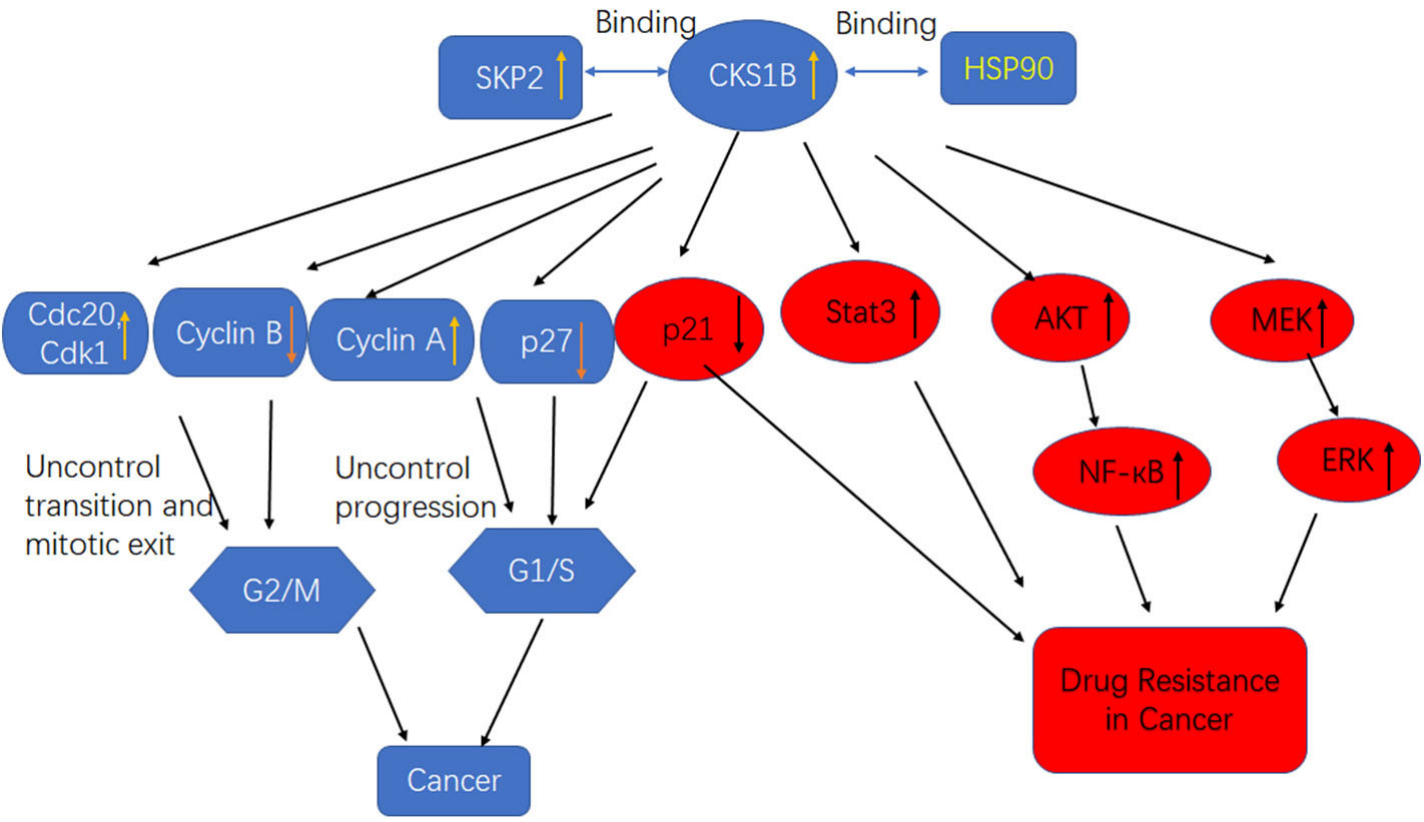
High CKS1B promotes cancer progression or cancer drug resistance through multiple pathways.

The CKS1 protein, which is encoded by the human CKS1B gene, consists of 79 amino acids and is a member of the CKS/SUC1 protein family. CKS1 can regulate the mitotic cycle in eukaryotes (26). In mammalian cells, P27 and P21 control the cell cycle from G1 phase to S phase and are essential proteins for phase transformation. The P27kip1 protein, a member of the CIP/kip family, inhibits most CDKs and arrests the cell cycle in S phase (27). CKS1B is an indispensable regulatory unit of the SCFskp^2^ ubiquitin ligase complex that can promote the binding of SCF to the cyclin inhibitor P27^Kip1^, after which phosphorylated P27 and p21 are recognized by the SCF/skp2 complex and catalyze multiple surface connections. The recognition and degradation of a ubiquitinated chain by the 26S protease promotes transition of the cell cycle from G1 to S phase (28, 29). CKS1B has three protein-binding sites, including an S-phase kinase-associated protein 2 (SKP2)-binding site and a CDK-binding site, the latter of which plays an important role in the ubiquitination process regulated by CKS1B (Figure 2). As a ubiquitin-regulating protein, CKS1B can specifically bind the SCF/SKP2 complex, which contains SKP1, Cullin1, F-box proteins, and the ubiquitin ligase E3-SKP2 (30).

## CKS1B IS A Drug Resistance-inducing Gene

Tumor resistance can be induced by many stimuli, among which gene mutation is of increasing concern. Studies showed that erlotinib, gefitinib, afatinib, and other epidermal growth factor receptor-tyrosine kinase inhibitors (EGFR-TKIs) could effectively improve the objective response rate and overall survival of NSCLC patients with EGFR mutations (including the deletion of exon 19 and the single point mutation L858R in exons 18-21). However, sustained EGFR-TKI treatment can lead to secondary resistance, and more 50% of secondary resistance is caused by the T790M secondary mutation in the catalytic pyrolysis region of the EGFR tyrosine kinase domain. In addition, HER2/neu (ErbB2), EGFR (ErbB1), HER3 (ErbB3), and HER4 (ErbB4) are members of the ErbB receptor-tyrosine kinase (RTK) family, and the oncogenic mutation patterns of EGFR and HER2 provide attractive options for targeted NSCLC therapy. Preclinical studies *in vitro* and *in vivo* models and human tissues have shown that overexpression of HER2 can lead to drug resistance to EGFR-TKIS and erlotinib resistance in mice and patients. Clinical studies have found that the current treatments for MM are ineffective in killing cells that specifically express CKS1B, leading to poor patient prognosis (23, 31). Studies have shown that overexpression of CKS1B promoted drug resistance in MM cells (31). In addition, *in vitro* proliferation experiments showed that CKS1 overexpression significantly enhanced the proliferation of liver cancer cells (15). Wang et al. observed that CKS1B overexpression in lung cancer cells achieved through lentiviral infection enhanced drug resistance by inhibiting cisplatin (CDDP)- and doxorubicin (DOX)-induced apoptosis, supporting the critical role of CKS1B in lung cancer progression (25). Furthermore, CKS1B upregulation is a predictor of poor prognosis and aggressive disease in many other malignancies (32–34). CKS1B has been identified as a ubiquitin-like protein system resistance gene that can selectively induce resistance to ubiquitin-like protein synthesis inhibitors but no other antitumor drugs. Furthermore, research has shown that CKS1B induces resistance to ubiquitin-like protein synthesis inhibitors such as bortezomib by inhibiting expression of the SCF/SKP2 substrate p21.

## Cks1B Activates Stat3 and Mek/Erk Through Skp2- and P27Kip1-independent Pathways

From cell surface receptors to transcription factors, members of the MEK/ERK signaling pathway, including the prosurvival proteins myeloid cell leukemia 1 and caspase-9, are involved in gene regulation (35) and protein activity regulation (36). Many diseases, such as cancer, human immunodeficiency virus infection (37), cardiac hypertrophy (38), and Parkinson's disease (39), result from aberrant regulation of the MEK/ERK signaling pathway. In particular, current research on the drugs used to treat cancer are focused on the ERK pathway (40). Moreover, a previous study demonstrated the potential of inactivating the MEK/ERK signaling pathway as a therapeutic target and an effective cancer treatment (41). Furthermore, the downregulation of CKS1B could inhibit the proliferation, migration, invasion, and angiogenesis of RB cells through inactivation of the MEK/ERK signaling pathway (14).

CKS1 is an important component of the SCF-Skp2 ubiquitin ligase complex, which degrades the CDK inhibitors p27Kip1, p21Cip1, and p130/Rb2. Interestingly, in addition to its influence on cell growth and survival through the regulation of p27Kip1, CKS1B leads to cell death in the presence of the p27Kip1 locus and inhibits the growth of MM cells. This observation indicates that CKS1B promotes cell proliferation independent of p27Kip1- and SKP2-associated mechanisms (16). Shi et al. determined that CKS1B upregulation activated the STAT3 and MEK/ERK pathways, whereas SKP2 knockdown or p27Kip1 overexpression activated, rather than suppressed, the STAT3 and MEK/ERK pathways, suggesting that the effects of SKP2 overexpression and p27 Kip1 inhibition on STAT3 and MEK/ERK were opposite those of CKS1B overexpression (Figure 2). Furthermore, MM cell death and growth inhibition induced by CKS1-1B knockout were partially eliminated by activation of the STAT3 and MEK/ERK/BCL2 signaling pathways (31). In addition, CKS1B downregulation inhibited RB cell proliferation, invasion, migration, and angiogenesis by blocking the MEK/ERK signaling pathway.

## Cks1B Induces Drug Resistance Through Targeting Heat Shock Protein 90 (Hsp90)

Previous studies showed that Hsp90 overexpression was induced in MM cells by the activation of STAT3 and MAPK signaling, which was essential for tumor cell survival. Overexpression of Hsp90 has been observed in many cancer types, and it is speculated that abnormal Hsp90 signaling must be maintained for the survival of malignant cells (42). Some components of the growth and survival pathways associated with tumor cells are Hsp90 clients, so Hsp90 is believed to maintain the functional expression of oncoproteins while enabling transformed cells to tolerate unbalanced signaling. These characteristics make Hsp90 a potential target for the development of anticancer drugs (43, 44). Wang et al. first reported that CKS1B uses the Hsp90 and MEK1/2 pathways in lung cancer cells to develop chemical resistance (Figure 2) (25). CKS1B upregulates Hsp90 independently through SKP2 and p27. In contrast, the Hsp90 inhibitor PU-H71 could inhibit the substrate protein AKT in CKS1B-overexpressing (OE) H358 cells, indicating that CKS1B mainly activates Hsp90 to resist CDDP. Furthermore, a proteomic study demonstrated that Cks1 could bind Hsp90 and its cochaperone in Ramos lymphoma cells (45).

## Status of Research on Cks1B Targeting in Cancer

### Use of Non-coding RNA to Target Cks1B for Cancer Treatment

#### Use of Mirnas to Target Cks1B for Cancer Treatment

MicroRNAs (miRNAs) are small non-coding RNA oligonucleotides ~21–23 nucleotides in length that can regulate genes at the posttranscriptional level with functions in various biological processes (46, 47). The dysregulation of miRNAs can lead to a variety of diseases, including developmental disorders, neurological diseases, and cancer. Abnormal miRNA and mRNA expression has been noted in many cancers (48–52). Therefore, a comprehensive analysis of miRNA and mRNA expression should be performed to enhance our understanding of miRNAs and mRNAs during tumorigenesis. Many databases and target prediction tools have recently been used to identify potential targets for miRNAs. However, the identification of miRNA targets is faced with enormous challenges.

Some studies have used bioinformatics methods to screen miRNAs that target CKS1B via TargetScan 7.2 and microRNA.org. In a bioinformatics analysis, several miRNAs targeting CKS1B (miR-125a, miR-1258, miR-197, miR-181a-1, miR-361, and miR-485) were predicted. Among mimics of these miRNAs, miR-1258 mimics inhibited the mRNA and protein expression of CKS1B in colorectal cancer (CRC) cells compared to cells treated with only negative control (NC) mimics. In addition, experiments have shown that miR-1258 exerts a tumor-suppressive effect through immediately downregulation of the oncogenic CKS1B gene in CRC (24). Other studies have shown that miR-204 negatively regulates the expression of CKS1B in gastric cancer (53). Yu Fujita found that miR-197 was downregulated in platinum-resistant NSCLC specimens, leading to *in vitro* and *in vivo* chemical resistance, tumorigenicity, and increased lung metastasis. Mechanistic studies have shown that the miR-197-mediated CKS1B/STAT3 axis plays a role in tumor progression and is regulated by multiple oncogenes (Bcl-2, c-Myc, and cyclin D1) and that PD-L1 is a putative biomarker of this axis. In addition, we demonstrated that miR-197 mimics could cause highly resistant PD-L1 cells to become sensitive to chemotherapy. Ting La's study showed that miR-27b-3p targets CKS1B, resulting in a decrease in p27Kip1 mediation by Skp2. The coding region for miRNA-27b-3p is embedded in an intron spanning the open reading frames of three genes on chromosome 9 and is activated by p53 transcription. The results of Ting La's study showed that miRNA-27b-3p is an important regulator of cancer cell dormancy in response to p53 and suggest that the manipulation of miRNA-27b-3p may represent a new therapeutic approach to improve cancer treatment outcomes (54).

#### Use of Long Non-coding RNAs (Lncrnas) Targeting CKS1B for Cancer Treatment

LncRNAs, which are present at lower cellular concentrations and exhibit higher tissue specificity than protein-encoding RNAs, participate in cell differentiation, proliferation, and apoptosis under different conditions. Furthermore, the differential expression of several lncRNAs shown in subjects with cancer suggests that lncRNAs can be used as biomarkers in the treatment of malignant tumors (55, 56). Metastasis-associated lung adenocarcinoma transcript 1 (MALAT1) is a novel lncRNA that was initially found to be overexpressed in patients with NSCLC at high risk for metastasis (57). The upregulation of MALAT1 is related to cancer cell proliferation and metastasis and the malignant development of esophageal squamous cell carcinoma (ESCC) (58, 59). MALAT1 has even been suggested as a predictor of poor prognosis in patients with midthoracic ESCC who have undergone radical resection (57, 60). In addition, experiments have revealed that CKS1 expression is positively regulated by MALAT1. This study not only elaborated on the function of the MALAT1/CKS1 pathway in regulating radiosensitivity in ESCC but also suggests a new adjunct strategy to increase the efficacy of radiation therapy in the treatment of ESCC (61).

### Use of Natural Medicines Targeting Cks1B for Tumor Treatment

Due to their low selectivity, radiotherapy and drug therapies currently used for clinical cancer treatment not only damage tumor cells but also kill healthy cells to varying degrees. Furthermore, they elicit many adverse reactions, and their long-term use leads to drug resistance. The development of drug resistance is one of the causes of chemotherapy failure. Therefore, much research has been devoted to identifying natural active antitumor ingredients from various organisms and natural compounds with antitumor activity. 3-O-(Z)-coumaric acid (3-COA), an oleanolic acid and active ingredient in the leaves of *Elaegnus oldhamii* Maxim, was shown to mimic the antitumor activity of a specific inhibitor of Hsp90 (PU-H71) against A549 lung cancer cells. In H358 CKS1b-OE cells and relapsed lung cancer, PU-H71 has antitumor activity alone or in combination with CDDP or DOX, which it achieves by inhibiting Hsp90. This observation suggests that 3-COA is a novel inhibitor of Hsp90. 3-COA, as a new type of antitumor drug, exhibited excellent antitumor activity alone or in combination with CDDP against chemically resistant or non-resistant lung cancer through the Hsp90/MEK signaling pathway. Therefore, 3-COA might be a candidate compound for reversing drug resistance in the clinical treatment of lung cancer (25).

### Nedd8 Inhibition Overcame Cks1B-Induced Drug Resistance in MM by Upregulating p21

MLN4924, a new and selective ubiquitin-like inhibitor with a structure similar to the energy-supply molecule ATP/AMP, is involved in the process of ubiquitin-like modification and selectively inhibits a ubiquitin-like activating enzyme (NEDD8-activating enzyme, NAE), thereby inhibiting the function of NEDD8 from a ubiquitin-like molecule-activating enzyme to a ubiquitin-like molecule-binding enzyme. Zhou et al. showed that CKS1B-OE cells were resistant to bortezomib but sensitive to MLN4924. Furthermore, the treatment of CKS1B-OE cells with MLN4924 reduced cell proliferation and clone formation and induce senescence by upregulating p21 in MM (23).

## Conclusion

As is a member of the Cks/Suc1 family of proteins, CKS1B plays an important role in the regulation of cell cycle progression. Increased CKS1B expression is involved in tumor initiation, maintenance, and progression and positively correlated with poor prognosis. Furthermore, CKS1B accelerates chemotherapeutic resistance in many types of cancers, while a decrease in CKS1B makes these tumor cells sensitive to chemotherapeutic drugs, suggesting that CKS1B is a promising treatment target in cancers. Recently, researchers found some drugs that reverse tumor resistance; these drugs include miRNA- and lncRNA-based drugs, natural medicines, and ubiquitin-like inhibitors but have not undergone clinical trial. The molecular mechanism by which CKS1B interacts with its binding targets remains unknown. Thus, more work to further understand the primary mechanisms underlying the function of CKS1B and its regulation is needed.

## Author Contributions

YZ, XY, JX, WS, QH, and HW contributed equally to the design, analysis, and interpretation of data. YZ and HW drafted the paper. All authors read and approved the final manuscript.

## Conflict of Interest

The authors declare that the research was conducted in the absence of any commercial or financial relationships that could be construed as a potential conflict of interest.

## References

[1] Bray F Ferlay J Soerjomataram I Siegel RL Torre LA Jemal A. Global cancer statistics 2018: GLOBOCAN estimates of incidence and mortality worldwide for 36 cancers in 185 countries. CA Cancer J Clin. (2018) 68:394–424. 10.3322/caac.21492 30207593

[2] Torre LA Bray F Siegel RL Ferlay J Lortet-Tieulent J Jemal A. Global cancer statistics, 2012. CA Cancer J Clin. (2015) 65:87–108. 10.3322/caac.21262 25651787

[3] Lin A Wei T Meng H Luo P Zhang J. Role of the dynamic tumor microenvironment in controversies regarding immune checkpoint inhibitors for the treatment of non-small cell lung cancer (NSCLC) with EGFR mutations. J Mol Cancer. (2019) 18:139. 10.1186/s12943-019-1062-7 31526368 PMC6745797

[4] Taylor MD LaPar DJ Isbell JM Kozower BD Lau CL Jones DR. Marginal pulmonary function should not preclude lobectomy in selected patients with non-small cell lung cancer. J Thorac Cardiovasc Surg. (2014) 147:738–44. 10.1016/j.jtcvs.2013.09.064 24252944

[5] Krishnan A Nair SA Pillai MR. Loss of cks1 homeostasis deregulates cell division cycle. J Cell Mol Med. (2010) 14:154–64. 10.1111/j.1582-4934.2009.00698.x 19228269 PMC3837597

[6] Lan Y Zhang Y Wang J Lin C Ittmann MM Wang F. Aberrant expression of Cks1 and Cks2 contributes to prostate tumorigenesis by promoting proliferation and inhibiting programmed cell death. Int J Cancer. (2008) 123:543–51. 10.1002/ijc.23548 18498131 PMC3262990

[7] Hadwiger JA Wittenberg C Mendenhall MD Reed SI. The *Saccharomyces cerevisiae* CKS1 gene, a homolog of the *Schizosaccharomyces pombe* suc1+ gene, encodes a subunit of the Cdc28 protein kinase complex. J Mol Cell Biol. (1989) 9:2034–41. 10.1128/MCB.9.5.2034 2664468 PMC362996

[8] Hayles J Beach D Durkacz B Nurse P. The fission yeast cell cycle control gene cdc2: isolation of a sequence suc1 that suppresses cdc2 mutant function. Mol Gen Genet. (1986) 202:291–3. 10.1007/BF00331653 3010051

[9] Richardson HE Stueland CS Thomas J Russell P Reed SI. Human cDNAs encoding homologs of the small p34Cdc28/Cdc2-associated protein of *Saccharomyces cerevisiae* and *Schizosaccharomyces pombe*. J Genes Dev. (1990) 4:1332–44. 10.1101/gad.4.8.1332 2227411

[10] Tsai YS Chang HC Chuang LY Hung WC. RNA silencing of Cks1 induced G2/M arrest and apoptosis in human lung cancer cells. J IUBMB Life. (2005) 57:583–9. 10.1080/15216540500215531 16118116

[11] Basi G Draetta G. p13suc1 of *Schizosaccharomyces pombe* regulates two distinct forms of the mitotic cdc2 kinase. J Mol Cell Biol. (1995) 15:2028–36. 10.1128/MCB.15.4.2028 7891698 PMC230430

[12] Hindley J Phear G Stein M Beach D. Sucl+ encodes a predicted 13-kilodalton protein that is essential for cell viability and is directly involved in the division cycle of *Schizosaccharomyces pombe*. J Mol Cell Biol. (1987) 7:504-11. 10.1128/MCB.7.1.504 3031478 PMC365094

[13] Stella F Pedrazzini E Baialardo E Fantl DB Schutz N Slavutsky I. Quantitative analysis of CKS1B mRNA expression and copy number gain in patients with plasma cell disorders. J Blood Cells Mol Dis. (2014) 53:110–7. 10.1016/j.bcmd.2014.05.006 24973170

[14] Zeng Z Gao ZL Zhang ZP Jiang HB Yang CQ Yang J. Downregulation of CKS1B restrains the proliferation, migration, invasion and angiogenesis of retinoblastoma cells through the MEK/ERK signaling pathway. Int J Mol Med. (2019) 44:103–14. 10.3892/ijmm.2019.4183 31115482 PMC6559318

[15] Lee EK Kim DG Kim JS Yoon Y. Cell-cycle regulator Cks1 promotes hepatocellular carcinoma by supporting NF-κB-dependent expression of interleukin-8. J Cancer Res. (2011) 71:6827–35. 10.1158/0008-5472.CAN-10-4356 21917729

[16] Zhan F Colla S Wu X Chen B Stewart JP Kuehl WM. CKS1B, overexpressed in aggressive disease, regulates multiple myeloma growth and survival through SKP2- and p27Kip1-dependent and -independent mechanisms. J Blood. (2007) 109:4995–5001. 10.1182/blood-2006-07-038703 17303695 PMC1885527

[17] Wang X Xu J Ju S Ni H Zhu J Wang H. Livin gene plays a role in drug resistance of colon cancer cells. J Clin Biochem. (2010) 43:655–60. 10.1016/j.clinbiochem.2010.02.004 20171199

[18] Zolota VG Tzelepi VN Leotsinidis M Zili PE Panagopoulos ND Dougenis D. Histologic-type specific role of cell cycle regulators in non-small cell lung carcinom. J Surg Res. (2010) 164:256–65. 10.1016/j.jss.2009.03.035 19691991

[19] Kitajima S Kudo Y Ogawa I Bashir T Kitagawa M Miyauchi M. Role of Cks1 overexpression in oral squamous cell carcinomas: cooperation with Skp2 in promoting p27 degradation. J Am J Pathol. (2004) 165:2147–55. 10.1016/S0002-9440(10)63264-6 15579456 PMC1618711

[20] Slotky M Shapira M Ben-Izhak O Linn S Futerman B Tsalic M. The expression of the ubiquitin ligase subunit Cks1 in human breast cancer. J Breast Cancer Res. (2005) 7:R737–44. 10.1186/bcr1278 16168119 PMC1242136

[21] Shaughnessy JJ Zhan F Burington BE Huang Y Colla S Hanamura I. A validated gene expression model of high-risk multiple myeloma is defined by deregulated expression of genes mapping to chromosome *1*. J Blood. (2007) 109:2276–84. 10.1182/blood-2006-07-038430 17105813

[22] Chen MH Qi C Reece D Chang H. Cyclin kinase subunit 1B nuclear expression predicts an adverse outcome for patients with relapsed/refractory multiple myeloma treated with Bortezomib. J Hum Pathol. (2012) 43:858–64. 10.1016/j.humpath.2011.07.013 22047644

[23] Huang J Zhou Y Thomas GS Gu Z Yang Y Xu H. NEDD8 inhibition overcomes CKS1B-induced drug resistance by upregulation of p21 in multiple myeloma. J Clin Cancer Res. (2015) 21:5532–42. 10.1158/1078-0432.CCR-15-0254 26156395 PMC4804624

[24] Hwang JS Jeong EJ Choi J Lee YJ Jung E Kim SK. MicroRNA-1258 inhibits the proliferation and migration of human colorectal cancer cells through suppressing CKS1B expression. J Genes. (2019) 10:110912. 10.3390/genes10110912 31717435 PMC6896137

[25] Wang H Sun M Guo J Ma L Jiang H Gu L. 3-O-(Z)-coumaroyloleanolic acid overcomes Cks1b-induced chemoresistance in lung cancer by inhibiting Hsp90 and MEK pathways. Biochem Pharmacol. (2017) 135:35–49. 10.1016/j.bcp.2017.03.007 28288818

[26] Egan EA Solomon MJ. Cyclin-stimulated binding of Cks proteins to cyclin-dependent kinases. J Mol Cell Biol. (1998) 18:3659–67. 10.1128/MCB.18.7.3659 9632748 PMC108948

[27] Martinsson-Ahlzen HS Liberal V Grunenfelder B Chaves SR Spruck CH Reed SI. Cyclin-dependent kinase-associated proteins Cks1 and Cks2 are essential during early embryogenesis and for cell cycle progression in somatic cells. J Mol Cell Biol. (2008) 28:5698–709. 10.1128/MCB.01833-07 18625720 PMC2546922

[28] Ganoth D Bornstein G Ko TK Larsen B Tyers M Pagano M. The cell-cycle regulatory protein Cks1 is required for SCF(Skp2)-mediated ubiquitinylation of p27. J Nat Cell Biol. (2001) 3:321–4. 10.1038/35060126 11231585

[29] Spruck C Strohmaier H Watson M Smith AP Ryan A Krek TW. A CDK-independent function of mammalian Cks1: targeting of SCF(Skp2) to the CDK inhibitor p27Kip1. Mol Cell. (2001) 7:639–50. 10.1016/S1097-2765(01)00210-6 11463388

[30] Pines J. Cell cycle: reaching for a role for the Cks proteins. J Curr Biol. (1996) 6:1399–402. 10.1016/S0960-9822(96)00741-5 8939596

[31] Shi L Wang S Zangari M Xu H Cao TM Xu C. Over-expression of CKS1B activates both MEK/ERK and JAK/STAT3 signaling pathways and promotes myeloma cell drug-resistance. J Oncotarget. (2010) 1:22–33. 10.18632/oncotarget.105 20930946 PMC2949973

[32] Kawakami K Enokida H Tachiwada T Nishiyama K Seki N Nakagawa M. Increased SKP2 and CKS1 gene expression contributes to the progression of human urothelial carcinoma. J Urol. (2007) 178:301–7. 10.1016/j.juro.2007.03.002 17499794

[33] Liu Z Fu Q Lv J Wang F Ding K. Prognostic implication of p27Kip1, Skp2 and Cks1 expression in renal cell carcinoma: a tissue microarray study. J Exp Clin Cancer Res. (2008) 27:51. 10.1186/1756-9966-27-51 18922157 PMC2579281

[34] Shapira M Ben-Izhak O Slotky M Goldin O Lahav-Baratz S Hershko DD. Expression of the ubiquitin ligase subunit cyclin kinase subunit 1 and its relationship to S-phase kinase protein 2 and p27Kip1 in prostate cancer. J Urol. (2006) 176:2285–9. 10.1016/j.juro.2006.07.051 17070313

[35] Zassadowski F Rochette-Egly C Chomienne C Cassinat B. Regulation of the transcriptional activity of nuclear receptors by the MEK/ERK1/2 pathway. Cell Signal. (2012) 24:2369–77. 10.1016/j.cellsig.2012.08.003 22906493

[36] Gao N Budhraja A Cheng S Liu EH Huang C Chen J. Interruption of the MEK/ERK signaling cascade promotes dihydroartemisinin-induced apoptosis *in vitro* and *in vivo*. Apoptosis. (2011) 16:511–23. 10.1007/s10495-011-0580-6 21336837

[37] Lieske NV Tonby K Kvale D Dyrhol-Riise AM Tasken K. Targeting tuberculosis and HIV infection-specific regulatory T cells with MEK/ERK signaling pathway inhibitors. PLoS ONE. (2015) 10:e0141903. 10.1371/journal.pone.0141903 26544592 PMC4636186

[38] Ren J Zhang N Liao H Chen S Xu L Li J. Caffeic acid phenethyl ester attenuates pathological cardiac hypertrophy by regulation of MEK/ERK signaling pathway *in vivo* and *in vitro*. Life Sci. (2017) 181:53–61. 10.1016/j.lfs.2017.04.016 28449869

[39] Liu C Lee WC Huang BM Chia YC Chen YC Chen Y. 16-Hydroxycleroda-3 C, 13-dien-15, 16-olide inhibits the proliferation and induces mitochondrial-dependent apoptosis through Akt, mTOR, and MEK-ERK pathways in human renal carcinoma cells. Phytomedicine. (2017) 36:95–107. 10.1016/j.phymed.2017.09.021 29157834

[40] Xie C Li Y Li LL Fan XX Wang YW Wei CL. Identification of a new potent inhibitor targeting KRAS in non-small cell lung cancer cells. Front Pharmacol. (2017) 8:823. 10.3389/fphar.2017.00823 29184501 PMC5694459

[41] Cyprian FS Al-Farsi HF Vranic S Akhtar S Al Moustafa EA. Epstein-barr virus and human papillomaviruses interactions and their roles in the initiation of epithelial-mesenchymal transition and cancer progression. Front Oncol. (2018) 8:111. 10.3389/fonc.2018.00111 29765906 PMC5938391

[42] Whitesell L Lindquist SL. HSP90 and the chaperoning of cancer. J Nat Rev Cancer. (2005) 5:761–72. 10.1038/nrc1716 16175177

[43] Workman P. Combinatorial attack on multistep oncogenesis by inhibiting the Hsp90 molecular chaperone. J Cancer Lett. (2004) 206:149–57. 10.1016/j.canlet.2003.08.032 15013520

[44] Zhang H Burrows F. Targeting multiple signal transduction pathways through inhibition of Hsp90. J Mol Med. (2004) 82:488–99. 10.1007/s00109-004-0549-9 15168026

[45] Khattar V Fried J Xu B Thottassery JV. Cks1 proteasomal degradation is induced by inhibiting Hsp90-mediated chaperoning in cancer cells. Cancer Chemother Pharmacol. (2015) 75:411–20. 10.1007/s00280-014-2666-7 25544127

[46] Tomczak K Czerwinska P Wiznerowicz M. The Cancer Genome Atlas (TCGA): an immeasurable source of knowledge. J Contemp Oncol. (2015) 19:A68–77. 10.5114/wo.2014.47136 25691825 PMC4322527

[47] Deng M Bragelmann J Schultze JL Perner S. Web-TCGA: an online platform for integrated analysis of molecular cancer data sets. J BMC Bioinform. (2016) 17:72. 10.1186/s12859-016-0917-9 26852330 PMC4744375

[48] Guo WG Zhang Y Ge D Zhang YX Lu CL Wang Q. Bioinformatics analyses combined microarray identify the desregulated microRNAs in lung cancer. J Eur Rev Med Pharmacol Sci. (2013) 17:1509–16. 23771539

[49] Song F Yang D Liu B Guo Y Zheng H Li L. Integrated microRNA network analyses identify a poor-prognosis subtype of gastric cancer characterized by the miR-200 family. J Clin Cancer Res. (2014) 20:878–89. 10.1158/1078-0432.CCR-13-1844 24352645

[50] Lin L Lin Y Jin Y Zheng C. Microarray analysis of microRNA expression in liver cancer tissues and normal control. J Gene. (2013) 523:158–60. 10.1016/j.gene.2013.02.055 23583794

[51] Feng J Huang C Diao X Fan M Wang P Xiao Y. Screening biomarkers of prostate cancer by integrating microRNA and mRNA microarrays. J Genet Test Mol Biomarkers. (2013) 17:807–13. 10.1089/gtmb.2013.0226 23984644 PMC3816787

[52] Shrestha S Hsu SD Huang WY Huang HY Chen W Weng SL. A systematic review of microRNA expression profiling studies in human gastric cancer. J Cancer Med. (2014) 3:878–88. 10.1002/cam4.246 24902858 PMC4303155

[53] Shrestha S Yang CD Hong HC Chou CH Tai CS Chiew MY. Integrated microRNA-mRNA analysis reveals mir-204 inhibits cell proliferation in gastric cancer by targeting CKS1B, CXCL1, and GPRC5A. Int J Mol Sci. (2017) 19:87. 10.3390/ijms19010087 29283424 PMC5796037

[54] La T Liu GZ Farrelly M Cole N Feng YC Zhang YY. A p53-responsive miRNA network promotes cancer cell quiescence. Cancer Res. (2018) 78:6666–79. 10.1158/0008-5472.CAN-18-1886 30301840

[55] Sugihara H Ishimoto T Miyake K Izumi D Baba Y Yoshida N. Noncoding RNA expression aberration is associated with cancer progression and is a potential biomarker in esophageal squamous cell carcinoma. Int J Mol Sci. (2015) 16:27824–34. 10.3390/ijms161126060 26610479 PMC4661918

[56] Lin CY Xu HM. Novel perspectives of long non-coding RNAs in esophageal carcinoma. Carcinogenesis. (2015) 36:1255–62. 10.1093/carcin/bgv136 26392258

[57] Cao X Zhao R Chen Q Zhao Y Zhang B Zhang Y. MALAT1 might be a predictive marker of poor prognosis in patients who underwent radical resection of middle thoracic esophageal squamous cell carcinoma. Cancer Biomark. (2015) 15:717–23. 10.3233/CBM-150513 26406400 PMC12965489

[58] Wang W Zhu Y Li S Chen X Jiang G Shen Z. Long noncoding RNA MALAT1 promotes malignant development of esophageal squamous cell carcinoma by targeting beta-catenin via Ezh2. J Oncotarget. (2016) 7:25668–82. 10.18632/oncotarget.8257 27015363 PMC5041935

[59] Peng Y-T Wu W-R Chen L-R Kuo K-K Tsai C-H Huang Y-T. Upregulation of cyclin-dependent kinase inhibitors CDKN1B and CDKN1C in hepatocellular carcinoma-derived cells via goniothalamin-mediated protein stabilization and epigenetic modifications. J Toxicol Rep. (2015) 2:322–32. 10.1016/j.toxrep.2015.01.010 28962365 PMC5598353

[60] Ji P Diederichs S Wang W Boing S Metzger R Schneider PM. MALAT-1, a novel noncoding RNA, and thymosin beta4 predict metastasis and survival in early-stage non-small cell lung cancer. J Oncogene. (2003) 22:8031–41. 10.1038/sj.onc.1206928 12970751

[61] Li Z Zhou Y Tu B Bu Y Liu A Kong J. Long noncoding RNA MALAT1 affects the efficacy of radiotherapy for esophageal squamous cell carcinoma by regulating Cks1 expression. J Oral Pathol Med. (2017) 46:583–90. 10.1111/jop.12538 27935117

